# Clinical utility of procalcitonin in severe odontogenic maxillofacial infection

**DOI:** 10.1186/s40902-020-00288-x

**Published:** 2021-01-09

**Authors:** Ji-Kwan Kim, Jae-Hoon Lee

**Affiliations:** Department of Oral and Maxillofacial Surgery, College of Dentistry, Dankook University, 119 Dandae-ro, Dongnam-gu, Cheonan, Chungnam South Korea

**Keywords:** Abscess, Head and neck infection, Dental infections, Procalcitonin, Sepsis, SIRS

## Abstract

**Background:**

Most of the maxillofacial infections are bacterial infections, and there is a possibility that systemic infections occur by maxillofacial infections. The aim of this study was to investigate the diagnostic value of procalcitonin in patients with odontogenic bacterial infections of the maxillofacial region.

**Methods:**

We enrolled sixty patients, who were admitted with odontogenic maxillofacial infection from September 2018 to March 2020. White blood cell counts, C-reactive protein, and procalcitonin concentrations were evaluated. Sixty patients were classified into two groups, sepsis and non-sepsis groups, based on systemic inflammatory response syndrome. A Student *t* test was performed to statistically analyze the difference in inflammatory markers between sepsis and non-sepsis groups.

**Results:**

The mean procalcitonin values on admission were 7.24 ng/mL (range, 0.09–37.15 ng/mL) and 0.40 ng/mL (range, 0.02–4.94 ng/mL) in the sepsis group and non-sepsis group, respectively. The procalcitonin values between the two groups showed a significant difference (*P* < 0.05). The area under the curve of procalcitonin was 0.927 (*P* < 0.001), and the cutoff value of procalcitonin that maximizes the area under the curve was calculated to be 0.87 ng/mL.

**Conclusions:**

According to our study, routine laboratory tests have insufficient accuracy in diagnosing sepsis syndrome. Therefore, it is strongly recommended to perform the procalcitonin test in patients with maxillofacial infection in addition to the conventional laboratory tests to diagnose the systemic inflammatory condition of the patients.

## Backgrounds

Maxillofacial infections are life-threatening diseases commonly caused by odontogenic origin. Fascial space infections of odontogenic origin have latent spaces that are anatomically connected to each other, thus allowing the infections to spread, causing airway compromise, and invading sensitive anatomical structures such as the mediastinum and spinal cord. It can spread to the area, be life threatening, and progress to sepsis due to bacterial infection in the blood.

Sepsis syndrome is a systemic inflammatory response syndrome (SIRS) that is triggered when blood is infected by a microorganism that has invaded the human body and is caused by the interaction between the inflammatory factor of the microorganism and the inflammatory response of the host. SIRS is the initial step in the systemic host response to infection or injury, defined to occur in patients with any two or more of the four clinical criteria listed in Table 1 [1]. Sepsis syndrome is defined as SIRS that can identify the source of infection and is caused by the interaction of microbial inflammatory factors with host inflammatory responses.

**Table 1:** Systemic inflammatory response syndrome criteria

Criterion	Positive result
Temperature	< 36 °C or > 38 °C
Pulse rate	> 90 beats per minute
Respiratory rate	> 20 breaths per minute
White blood cell count	< 4 or > 12 × 10^9^/L

Procalcitonin (PCT) is a precursor of calcitonin and is synthesized by thyroid C cells. Additionally, PCT is produced by the reaction to endotoxins and the mediation produced in response to bacterial infections [2]. PCT has the highest sensitivity and specificity for predicting systemic bacterial inflammation [3–5]. PCT levels are associated with the severity of bacterial infections and may also be helpful in determining the initiation and duration of antibiotic treatment via PCT measurements [6–8]. PCT analysis can be performed within 1 h, so that the patient’s systemic infection status can be quickly identified, initiating treatment.

There have been several studies assessing the utility of PCT analysis for systemic bacterial infections; however, only a few studies have reported on the utility of PCT in patients with odontogenic maxillofacial infections. Additionally, it is generally known that sepsis syndrome may occur due to severe odontogenic maxillofacial infection, although there have been no studies of patients with sepsis syndrome caused by odontogenic infection. This study aimed to investigate the diagnostic value of PCT in patients with odontogenic bacterial infections of the maxillofacial region.

## Materials and methods

### Patients

We studied patients who were admitted to the Department of Oral and Maxillofacial Surgery at Dankook University Hospital (Cheonan, Korea) from September 2018 to March 2020 for the treatment of odontogenic maxillofacial infections. Ethical approval was sought and given by the Dankook University Dental Hospital Institutional Review Board (IRB number: DKUH IRB 2020-05-006) for carrying out this study.

We collected details on age, sex, vital signs, underlying diseases, and site of infection. Adult patients (ages older than 19 years) were only included in the investigation, and patients with non-maxillofacial infections such as pneumonia and endocarditis were excluded.

The criteria for admission were swelling of the face or neck that suggested an abscess or cellulitis and manifestation of one or more of the following symptoms: a compromised airway, restricted mouth opening, dysphagia, or body temperature greater than 38 °C.

Antimicrobial drugs were administered from the day of admission to the day before discharge, and the abscess was drained. When deep space infection was observed or intraoral incision was difficult to perform due to severe restriction of mouth opening, drainage was performed under general anesthesia.

### Laboratory examination

Antecubital venous blood was drawn before admission for the measurement of white blood cell (WBC) counts, C-reactive protein (CRP), and PCT concentrations. Additionally, bloodstream infection was confirmed through blood culture tests in all patients. Simultaneously with the operation, pus culture was performed.

### Systemic inflammatory response syndrome analysis

The vital signs of patients and WBC were measured at the time of admission. Patients with any two or more of the following clinical criteria have been defined as SIRS: the body temperature less than 36 °C or more than 38 °C, the pulse rate over 90 times per minute, the respiratory rate over 20 times per minute, and the number of white blood cells less than 4 × 10^9^/L or more than 12 × 10^9^/L (Table 1). Patients with SIRS were classified in the sepsis group, and patients without SIRS were classified in the non-sepsis group.

### Diagnosis of maxillofacial fascial infection and severity scoring

The site of infection was confirmed via enhanced computed tomography taken at the time of admission, and the severity of infection was also recorded. The severity of infection was measured by the sum of each severity score of anatomic spaces affected by abscess or cellulitis based on the study by Flynn et al. [9]. Abscess with low risk to airway or vital structures such as vestibular, subperiosteal, infraorbital, and buccal space abscess was calculated as severity score of 1 point. Submandibular, submental, sublingual, pterygomandibular, submasseteric, superficial temporal, and deep temporal space abscess was calculated as severity score of 2 points which were considered moderate risk to airway or vital structures. At last, abscess with high risk to airway or vital structures such as lateral pharyngeal, retropharyngeal, pretracheal, and mediastinal space abscess was calculated as severity score of 3 points.

### Data analysis

After confirming SIRS, patients were classified based on the presence or absence of sepsis syndrome, and Student’s *t* test was performed to statistically analyze the difference in inflammatory markers between the two groups (the sepsis and non-sepsis groups). Second, to confirm that the degree of local site infection may affect sepsis syndrome, the severity score and the number of affected spaces were compared using Student’s *t* test.

Finally, to measure the sensitivity, specificity, and predictive cutoff value of PCT in diagnosing sepsis syndrome in patients with odontogenic maxillofacial infection, the receiver operating characteristic (ROC) curve was used to measure the area under the curve (AUC).

Statistical analysis was performed using the Statistical Package for the Social Sciences statistics software version 26 (IBM, Chicago, IL, USA). Data are expressed as the mean ± standard deviation (SD). *P* value less than 0.05 was considered as statistically significant.

## Results

From September 2018 to March 2020, we investigated sixty patients with odontogenic maxillofacial infections who were admitted to the Department of Oral and Maxillofacial Surgery at Dankook University Hospital (Cheonan, Korea). Patient ages ranged from 20 to 88 years (mean age = 56.04 years, SD = 18.15), with 60% being male (36) and 40% being female (24). The number of patients with diabetes mellitus was fourteen.

### Demographics of the sepsis and non-sepsis groups

The temperature, respiratory rate, heart rate, and WBC measured upon admission were applied to the criteria in Table 1 [1] to classify patients into the sepsis and non-sepsis groups. Of the sixty patients, forty-two patients were in the non-sepsis group and eighteen patients were in the sepsis group (Table 2). The average ages of the sepsis and non-sepsis groups were 58.1 years (range, 34–82 years) and 55.7 years (range, 20–88 years), respectively, and there was no statistically significant difference between the two groups (*P* > 0.05).

**Table 2:** Demographics; procalcitionin, C-reactive protein, and white blood cell level on admission; number of spaces involved; and severity score of the sepsis and non-sepsis groups

	Sepsis	Non-sepsis	*P* value
Sex			
Male	12	24	
Female	6	18	
Age (years)	58.06 ± 14.30 (34–82)	55.69 ± 19.69 (20–88)	
PCT level (ng/mL)	7.24 ± 9.20 (0.09–37.15)	0.40 ± 1.03 (0.02–4.94)	0.00
CRP level (mg/dL)	22.16 ± 9.02 (10.01–40.66)	10.83 ± 7.37 (1.15–28.12)	0.47
WBC level (× 10^3^/uL)	15.56 ± 6.61 (0.71–29.64)	13.92 ± 9.42 (7.01–18.84)	0.72
Number of involved spaces	2.50 ± 1.20 (1–5)	2.29 ± 1.33 (1–7)	0.93
Severity score	5.22 ± 2.69 (2–11)	4.33 ± 2.82 (1–14)	0.75

### Laboratory examination

The mean PCT values on admission were 7.24 ng/mL (range, 0.09–37.15 ng/mL) and 0.40 ng/mL (range, 0.02–4.94 ng/mL) in the sepsis group and non-sepsis group, respectively. PCT levels between the two groups were significantly different (*P* < 0.05). On the contrary, the CRP values were 22.16 mg/dL (range, 10.01–40.66) and 10.83 mg/dL (range, 1.15–28.12) in the sepsis group and the non-sepsis group, respectively, and the WBC values were 15.56 × 10^3^/uL (range, 0.71–29.64) and 13.92 × 10^3^/uL (range, 7.01–18.84), respectively. The CRP and WBC values did not show any significant difference between the two groups (Table 2).

According to the blood culture test results, only one patient was found to have bacterial infection, and blood culture test results from all other patients were negative. The bacterium detected was Gram-positive cocci *Peptostreptococcus micros*. This patient was admitted following the complaint of left submasseteric, pterygomandibular, superficial temporal, and infratemporal space abscess. The PCT level on admission was 9.66 ng/mL, and the CRP level was 36.04 mg/dL. According to her general condition (low blood pressure and high lactic acid levels), she was diagnosed with septic shock.

Based on pus culture test results, twenty-five patients had Gram-positive bacterial infection and seven patients had Gram-negative bacterial infection. The test results of the remaining twenty-eight patients were contaminated with more than three types of contaminated specimens. Among the sepsis group, Gram-positive bacteria were detected in nine patients, and three patients showed Gram-negative bacterial infection.

### Distribution of the involved space and severity score

Submasseteric, submandibular, and pterygomandibular spaces were the most affected fascial spaces (Table 3). The number of spaces involved in the sepsis group ranged from one to five, and the non-sepsis group ranged from one to seven. There was no significant difference in the number of spaces between the sepsis and non-sepsis groups (*P* = 0.93). As a result of calculating the severity score of infection, the sepsis and non-sepsis groups were 5.22 points (range, 2–11) and 4.33 points (range, 1–14), respectively. There was no significant difference in the severity score between the two groups (*P* = 0.75) (Table 2).

**Table 3:** Distribution of the involved fascial space

Fascial space	Sepsis	Non-sepsis	Total
Submasseteric	9	18	27 (19.4)
Submandibular	7	16	23 (16.5)
Pterygomandibular	7	14	21 (15.1)
Sublingual	4	12	16 (11.5)
Buccal	3	11	14 (10.1)
Submental	4	10	14 (10.1)
Infratemporal	5	3	8 (5.8)
Lateral pharyngeal	2	5	7 (5.0)
Superficial temporal	4	1	5 (3.6)
Canine	0	4	4 (2.9)
Total	45	94	139 (100)

## Determining the diagnostic value of procalcitonin

**Fig. 1:**
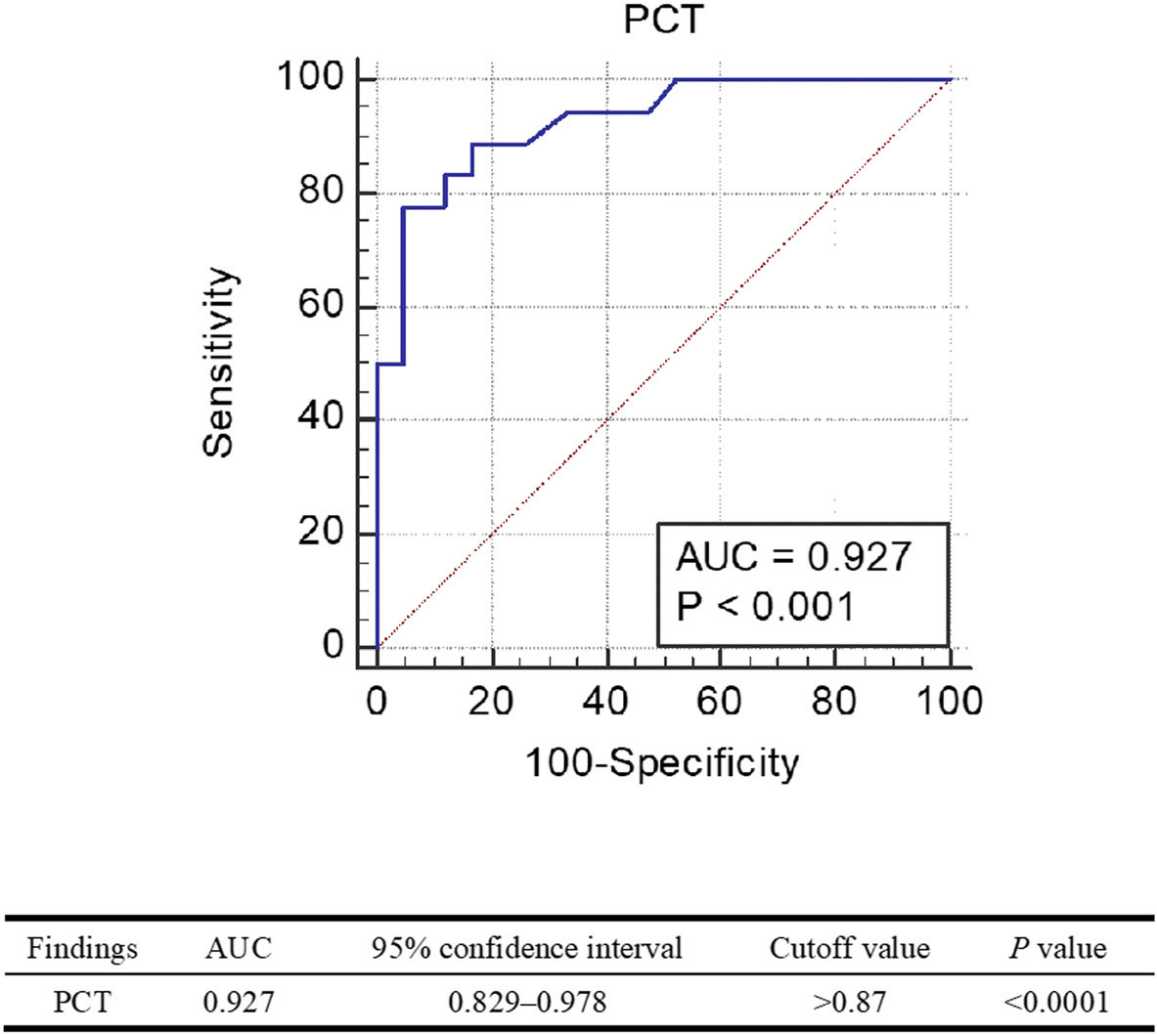
Sepsis syndrome receiver operating characteristic curves for procalcitonin test (95% confidence interval)

AUC values were measured using the ROC curve to confirm the diagnostic sensitivity and specificity of PCT levels in patients with sepsis syndrome among patients with maxillofacial infections. The AUC of PCT was 0.927 (Fig. 1) (*P* < 0.001), and PCT was confirmed to be a highly accurate test for diagnosing sepsis syndrome in patients with maxillofacial infections. The cutoff value of PCT that maximizes the AUC area was 0.87 ng/mL. At this point, the sensitivity and specificity were 77.78% and 95.24%, respectively (Fig. 1).

## Discussion

Maxillofacial infections have a potentially high risk of progressing to systemic infections. However, the traditional clinical signs of infection and routine laboratory tests for sepsis, such as CRP or WBC, are known to have insufficient diagnostic accuracy [10, 11]. Many studies have demonstrated that PCT levels are correlated with the severity of sepsis and could be used as a prognostic marker in patients with sepsis [12, 13]. Therefore, through this study, we sought to confirm the diagnostic value of PCT in patients with oral and maxillofacial infections.

Serum PCT concentration in healthy individuals is typically < 0.1 ng/mL [14]. Liaudat et al. studied PCT as an early marker of sepsis in a hospitalized patient population (8% prevalence of bacteremia) and found a high negative predictive value, depending on the cutoff value for the PCT level (99% for 0.2 ng/mL and 95% for 0.5 ng/mL) [15]. A low or normal PCT concentration does not always indicate the absence of bacterial infection. This may specifically be the case in the early course of a bacterial infection, or in localized infections. In the presence of bacterial infection, PCT increases, and the degree of increase correlates with the severity of the infection. Patients with PCT levels greater than 0.5 ng/mL are likely to have a systemic infection. Patients with localized infection have smaller increases in PCT compared to those with generalized sepsis, severe sepsis, and septic shock.

The advantage of the PCT test is that the results can be obtained quickly. Detection of bacterial infections in the blood is a key step in the diagnosis of sepsis and initiation of treatment with antimicrobials. However, culturing blood usually takes more than 48 h, and false-negative results are common. According to Cohen et al. [16], blood cultures are negative in 60 to 70% of patients with severe sepsis. In our study, among the sepsis syndrome patients, only one patient was confirmed to have a bloodstream bacterial infection. This result is most likely due to the antibiotic administered before the blood culture test in most patients. Scheer et al. [17] found that obtaining blood culture during antibiotic treatment can significantly reduce antigen detection, and blood culture should be performed prior to antibiotic administration. Early diagnosis of sepsis and administration of antibiotics is vital because progression to severe sepsis or septic shock has serious effects. Considering this perspective, traditional methods may overlook the appropriate timing of treatment. However, PCT is detectable 3 to 4 h following an infection, and it peaks at 6 to 12 h and has a half-life of approximately 24 h [18]. Treatment can be performed quickly based on the results of the PCT test.

Our study investigated whether PCT has better diagnostic utility in patients with odontogenic maxillofacial infections than other routine laboratory tests such as WBC or CRP. In our study, out of the sixty patients, eighteen patients (30%) had sepsis syndrome, and forty-two patients (70%) had no sepsis syndrome. Between the two groups, CRP and WBC values showed no significant differences, although the average PCT levels were 7.24 ng/mL in the sepsis group and 0.40 ng/mL in the non-sepsis group, and the difference was statistically significant (Table 2). There were no significant differences in both the number of affected spaces and the severity score between the sepsis and non-sepsis groups. The PCT level of one patient in the sepsis group was 22.68 ng/mL; however, only one space, the right sublingual space, was involved. That is, regardless of the severity of localized infection, systemic infection may occur, and PCT was the only statistically significant laboratory test between patients with systemic infection and localized infection. Similar results have been reported for localized maxillofacial cellulitis by Bertolus et al. [19], who reported that PCT showed almost low concentrations in patients with odontogenic maxillofacial cellulitis. In summary, the PCT level shows a low concentration when the infection in the maxillofacial region is limited to the local site and appears high when the infection spreads systemically.

We attempted to determine the cause of the increase in PCT levels in specific patients. In our study, patients who had infections in other areas except for the maxillofacial region were excluded, and none of the patients had chronic disease. Furthermore, PCT levels can be affected with diabetes mellitus. Wang et al. [20] reported that PCT levels in patients with diabetes mellitus were significantly higher than those in patients without diabetes mellitus. However, in our study, the number of patients with diabetes mellitus was fourteen, and there was no significant difference in PCT levels between patients with diabetes mellitus and patients without diabetes mellitus. We consider this result to be due to most of the glycemic control of diabetes mellitus patients performed normally.

Furthermore, we hypothesized that the bacterial species at the site of infection could influence the increase in PCT levels. There are several studies in which patients with sepsis have higher PCT levels for Gram-negative bacteria than for Gram-positive bacteria [21, 22]. Based on the results of pus culture, we classified the Gram-positive and Gram-negative groups and compared their PCT levels. The numbers of patients in the Gram-positive and Gram-negative groups were twenty-five and seven, respectively. There was no significant difference between the two groups. Moreover, a comparison was performed between the sepsis group. Among the sepsis group, nine and three patients had Gram-positive and Gram-negative bacterial infections, respectively. There was also no significant difference between the two groups. We hypothesize that this is due to the small number of sample size and the difficulty in performing accurate statistical analysis. Hence, further study on this topic is required.

Finally, we measured the accuracy of PCT in patients with maxillofacial infection using the ROC curve. The AUC of PCT was 0.927 (*P* < 0.001), and it was confirmed that PCT in patients with systemic inflammatory syndrome caused by odontogenic maxillofacial infection is highly accurate. Additionally, we evaluated the sensitivity and specificity of the conventional cutoff value (0.5 ng/mL) of PCT to diagnose sepsis. The sensitivity and specificity were 83.33% and 85.71%, respectively. On the contrary, the concentration (cutoff value) at the decisive point of PCT that maximizes the AUC area was 0.87 ng/mL. When the cutoff value was set to 0.87 ng/mL for sepsis syndrome, the sensitivity and specificity were 77.78% and 95.24%, respectively, and the false positive result was 0.071. The sensitivity was slightly lower than the cutoff value of 0.5 ng/mL; however, the specificity was significantly higher than other decision points (Table 4). Therefore, it is recommended to apply a cutoff value of 0.87 to diagnose sepsis syndrome in patients with maxillofacial infections. Additionally, among our patients, the number of patients with PCT levels greater than 10 ng/mL was three, which is known to have a high likelihood of severe bacterial sepsis or septic shock. All three patients were included in the sepsis group. Twelve patients had PCT levels ranging from 2 to 10 ng/mL. Among these patients, two were assigned in the non-sepsis group and the others were assigned in the sepsis group. This result is considered to be an error that occurred because the sensitivity and specificity of PCT were not 100%.

**Table 4:** Sensitivity and specificity as cutoff value of procalcitonin for the diagnosis of sepsis syndrome of maxillofacial infection

Cutoff value, ng/mL	Sensitivity, %	95% CI	Specificity, %	95% CI
> 0.40	83.33	58.6–96.4	83.33	68.6–93.0
> 0.50	83.33	58.6–96.4	85.71	71.5–94.6
**> 0.87**	**77.78**	**52.4–93.6**	**95.24**	**83.8–99.4**
> 2.00	72.22	46.5–90.3	95.24	83.8–99.4

## Conclusion

Odontogenic maxillofacial infections have a high potential to progress to systemic infections. According to our study, routine laboratory tests such as CRP and WBC have insufficient accuracy in diagnosing sepsis syndrome. Between the sepsis group and the non-sepsis group, CRP and WBC values had no significant difference. However, the average PCT levels were 7.24 ng/mL in the sepsis group and 0.40 ng/mL in the non-sepsis group, and there was a significant difference between the two groups. Additionally, regardless of the severity of localized infection, systemic infection may occur, and PCT was the only statistically significant laboratory test between patients with systemic infection and localized infection. Therefore, it is strongly recommended to perform the PCT test in patients with maxillofacial infection in addition to the conventional laboratory tests to diagnose the systemic inflammatory condition of the patients.

Additionally, according to our study, the AUC of PCT was 0.927 (*P* < 0.001), and PCT was confirmed to be a highly accurate test for diagnosing sepsis in patients with maxillofacial infections. The cutoff value of PCT that maximizes the AUC was 0.87 ng/mL. The sensitivity and specificity were 77.78% and 95.24%, respectively. Therefore, sepsis syndrome can be suspected when the PCT concentration of patients with maxillofacial infections is over 0.87 ng/mL.

## Data Availability

The datasets used and/or analyzed during the current study are available from the corresponding author on reasonable request.

## Abbreviations

SIRS: Systemic inflammatory response syndrome
PCT: Procalcitonin
WBC: White blood cell
CRP: C-reactive protein
ROC: Receiver operating characteristic
AUC: Area under the curve
